# c-MYC mRNA destabilization inhibited lethal pancreatic cancer *in vivo* with significant survival outcomes

**DOI:** 10.3389/fphar.2025.1630476

**Published:** 2025-08-29

**Authors:** Jigme P. Dorji, Queenie Chen, Sandali G. Perera, Fizza Aijaz, Petvy Li, Tanjina Sania, Hiroshi Matsui, Chidiebere U. Awah

**Affiliations:** ^1^ UTR Therapeutics Inc., New York, NY, United States; ^2^ Department of Chemistry, Hunter College, City University of New York, New York, NY, United States; ^3^ Ph.D. Program in Biochemistry, The Graduate Center, City University of New York, New York, NY, United States; ^4^ Department of Biochemistry, Weill Cornell Medicine, Cornell University, New York, NY, United States; ^5^ Ph.D. Program in Chemistry, The Graduate Center, City University of New York, New York, NY, United States

**Keywords:** pancreatic cancer, pancreatic ductal carcinoma, neuroendocrine pancreatic cancer, 3′UTRMYC1-18, c-Myc, PD-L1

## Abstract

Pancreatic ductal carcinoma is the most common and deadly form of pancreatic cancer, with an 11% survival rate. There is currently no cure. The first-line, mainstay therapy for pancreatic cancer is gemcitabine, capecitabine, or FOLFIRINOX. After 21 months, the chemoresistance begins, driven by the oncogenic c-MYC signal. This is a significant clinical and cancer biology challenge. The c-MYC oncogene has been shown to be overexpressed in primary (43.1%) and metastatic (31.6%) pancreatic cancers, respectively, and is the primary driver of the neoplastic changes and progression of pancreatic cancer metastasis. Here, we report the *in vivo* downregulation and inhibition of metastatic c-MYC-expressing lethal pancreatic cancer by the mRNA drug 3′UTRMYC1-18. The drug achieved on-target, *in vivo* c-MYC dose-dependent downregulation with complete pathological response, inhibition of liver, lung, and brain metastases with significant survival outcome, is safe, has a stable long half-life, and is well tolerated. Mechanistically, the therapeutic efficacy of the MYC-mRNA drug was achieved through downregulation of c-MYC-PD-L1.

## 1 Introduction

Pancreatic ductal carcinoma is the most common form of pancreatic cancer and is the deadliest, with a survival rate of 11% (Huang et al., 2021; Siegel et al., 2023). Lethal pancreatic cancer is extremely aggressive and metastasizes to the duodenum, liver, mediastinum, lungs, pericardium, and brain. The standard-of-care treatment for pancreatic cancer is gemcitabine, capecitabine, or FOLFIRINOX (Yao et al., 2022; Ko et al., 2012; Kindler et al., 2005; Kindler et al., 2010). Chemoresistance starts 21 months after this chemotherapy due to the upregulation of the c-MYC oncogenic signal. Approximately 43.1%–74.7% of the primary and metastatic drug-resistant pancreatic cancers express MYC (Schleger et al., 2002; Farrell et al., 2002; Sodir et al., 2020). This is a critical and significant challenge that must be addressed to improve pancreatic cancer clinical outcomes.

c-MYC is a basic helix-loop-helix transcription factor that binds the E-box sequences. This group of transcription factors is a superfamily comprising c-MYC, MYCN, and L-MYC (Conacci-Sorrell et al., 2014; Dhanaskeran and Deutzmann, 2022). The MYC is overexpressed in a variety of human cancers through amplification, insertion, and rearrangements. To date, there is no approved direct MYC inhibitor. To directly target MYC, we developed a destabilized c-MYC 3′UTR drug based on engineering the mRNA poly U stabilizing elements. The drug works by directly and specifically recognizing the endogenous c-MYC-mRNA exons. In turn, when the ribosomes try to translate this, the drug recognizes the destabilized elements and triggers the EXOSC4-PELO-RPL3 complex to degrade the destabilized MYC mRNA, leading to the downregulation of the MYC protein (Geisberg et al., 2014; Awah et al., 2024; Andersen and Jensen, 2015; Chonenberg and Maquat, 2012).

With this study, we have shown that the 3′UTRMYC1-18 drug is therapeutically effective both *in vitro* and *in vivo* in a titratable dose-dependent manner compared to the standard-of-care drugs in various MYC-driven lethal pancreatic cancers. The drug achieved long-term significant survival outcomes with survival benefits and inhibition of metastases. The drug was well tolerated at a low dose in this study, and in other studies that examined c-MYC-driven triple-negative breast cancer (TNBC), colon cancer, and ovarian cancer, the moderate and high doses were well tolerated without any blood cell dyscrasia or kidney, liver, pancreas, or gall bladder abnormalities.

Taken together, we present compelling evidence that the MYC-mRNA drug is effective as a monotherapy in *in vitro* and *in vivo* pancreatic cancer models using immunocompromised NSG mice. These data provide evidence that the MYC-mRNA drug will be an effective therapy for patients with lethal pancreatic cancer.

## 2 Materials and methods

### 2.1 The development of the 3′UTRMYC1-18 mRNA drug

We have previously described the development of the c-MYC-mRNA drug (Awah et al., 2024) and its mechanism of action. We discovered the stable mRNA poly U sequences on the 3′UTR of c-MYC and engineered them to unstable forms under the control of mRNA de-capping promoter DCP1A. The destabilized mRNA drug directly binds to its target mRNA recognition site either in-frame or at the 3′UTR or both and triggers the stalling of the ribosome. This stalling is sensed by the PELO-EXOSC4-RPL3 complex, which triggers the degradation of the target transcript. This process does not affect normal healthy cells because the healthy cells do not differentially express EXOSC4, PELO, or RPL3.

### 2.2 Cell culture

We obtained the following pancreatic cancer cells: PSN1 (RRID: CVCL_1644), MIA-Paca2 (RRID: CVCL_0428), PANC-1 (RRID: CVCL_0480), AC16 (RRID: CVCL_4U18), and RWPE1 (RRID: CVCL_3791) from ATCC. The PSN1 was grown in RPMI media supplemented with antibiotics/antimycotics before use. The PANC-1 and MIA-Paca-2 were grown in DMEM supplemented with antibiotics and anti-mycotics before use. FBS (10%) was added to the media. AC16 and RWPE1 were grown using the standard media purchased from ATCC. The cells were authenticated by ATCC by short-term repeat (STR) sequencing, and we regularly evaluated them for *mycoplasma* before use.

### 2.3 Dose-dependent IC50 determination and comparison with standard-of-care drugs

We determined the IC50 of the MYC-mRNA drug 3′UTRMYC1-18 by titrating the drug in a dose-dependent manner in a head-to-head comparison with the standard-of-care drugs (olaparib, paclitaxel, cisplatin, actinomycin D, bevacizumab, cyclophosphamide, MYCi975 (MYC-Max inhibitor), and osimertinib. All drugs were obtained from Selleckchem, USA. The doses used are from 2.5 µg to 40 µg. Serial dilutions of the drugs were made. We seeded the cells at 5,000 cells per well in a 96-well plate. The cells were allowed to attach for 24 h, and then the drugs were added. We incubated the treated cells for 72 h. We read the viability using the cell titre glo (Promega G7570) and normalized it to the controls. The data curves were fitted on the drug dose–response chart in GraphPad Prism (USA), and the IC50 values were derived.

### 2.4 Quantitative reverse transcription PCR

To quantify the dose-dependent downregulation of c-MYC transcript in pancreatic cancer, we treated the MIA-Paca-2 cells with a dose-dependent concentration of 3′UTRMYC1-18. We extracted the RNA using the Qiagen RNeasy kit (Cat No. 74104). The RNA was stored at −80°C before use. To reverse transcribe the RNA, we used the Superscript IV reverse transcriptase kit (Cat No. 18090200) to make cDNA. We designed the qPCR primers targeting the exons of MYC and GAPDH as a housekeeping gene control. All the primers used are described in ref 13. The delta CT was used to normalize the transcript expression.

### 2.5 Iron oxide nanocage and 3′UTRMYC mRNA drug complexation

The iron oxide nanocage and the MYC-mRNA drugs were complexed in a 1:1 ratio (Awah et al., 2024; Rampersaud and Fang, 2016), and the conjugates were incubated overnight before use at 4°C. Subsequently, the drug conjugate was found to be stable at room temperature, 4°C, and −20°C till use.

### 2.6 Animal study

**FIGURE 1:**
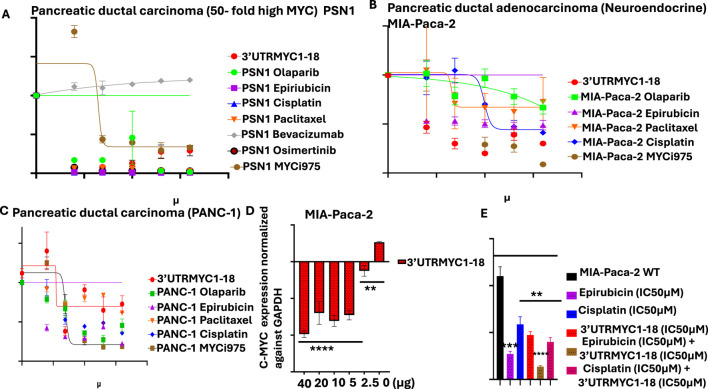
Determination of the IC50 of MYC-mRNA 3′UTRMYC1-18 and standard-of-care drugs, mRNA binding profiles, and combination dose. **(A)** The drug dose–response curve of 3′UTRMYC1-18 in a head-to-head comparison with the standard-of-care drugs and MYC-Max inhibitor in PSN1. **(B)** The drug dose–response curve of 3′UTRMYC1-18 in a head-to-head comparison with the standard-of-care drugs and MYC-Max inhibitor in MIA-Paca-2. **(C)** The drug dose–response curve of 3′UTRMYC1-18 in a head-to-head comparison with the standard-of-care drugs and MYC-Max inhibitor in PANC-1. **(D)** The bar charts show dose-dependent downregulation of c-MYC-mRNA expression in MIA-Paca-2 by 3′UTRMYC1-18. **(E)** The bar chart shows the viability of MIA-Paca-2 cells under the IC50 dose of 3′UTRMYC1-18 and standard-of-care drugs alone and in combination, normalized against the wild-type control. ****p < 0.00001, ***p = 0.0013, **p = 0.024, Two-tailed T-test.

We performed animal studies to validate the *in vivo* therapeutic efficacy of the c-MYC-mRNA drug in lethal pancreatic cancer. We obtained Institutional Animal Care and Use Committee (IACUC) institutional approval from the CUNY Institutional Review Board. We ordered five female and three male NSG mice aged 5–8 weeks and weighing 15–21 g, from the Jackson Laboratory. Once received, we allowed the mice to acclimatize according to the protocol. We implanted 10 million PSN1 cells into the flanks of the mice. Seven days post implantation, the tumor engrafted and, on day 10, we randomized the mice into a vector + nanocage group (N = 4) and a 3× IC50 3′UTRMYC1-18 + nanocage group (N = 4). All mice had tumors, and there were no excluded mice. We started treating the mice intravenously with the 3XIC50 dose of the drug, which is 3.5 µg (as determined from Figure 1), 2× per week. On day 25, we collected the blood of the mice to assess for the safety profile (full blood count, electrolyte, liver, kidney, pancreatic, and gall bladder function). The vector + nanocage-treated tumor-bearing mice died on day 26 at a pre-determined tumor volume of 0.8 cm^3^, upon which the animal was euthanized in 100% CO_2_. The 3× IC50 group lived till day 32.

To establish the dose-dependent inhibition of tumor volume by the MYC-mRNA drug in pancreatic cancer, we obtained eight mice (seven females, one male) from the Jackson Laboratory, age 5–8 weeks and weighing 15–20 g. We allowed the mice to acclimatize and implanted 10 million PSN1 cells into their flanks. By day 7, they were engrafted, and we randomized them into three groups based on tumor size and weight. The groups were 1) vector + nanocage (N = 2); 2) 6× IC50 21 µg (N = 3); 3) 9× IC50 31 µg (N = 3). We then dosed the mice 2× per week intravenously through the tail veins till day 28. By day 27, the vector + nanocage-treated mice had died. The 6× IC50-treated group died on day 33. We dosed the 9× IC50 1× per week from day 28 to day 33, after which we stopped dosing. The mice in the 9× IC50 group lived till day 55 and were euthanized. We recorded daily tumor volumes, weights, and body condition scores. The mice were euthanized with CO_2_ at the end of the study. The log-rank Mantel test was used to determine statistical significance between the *in vivo* treatment groups. The Kaplan–Meier survival curve was used to determine survival differences between the various treatment groups and controls. Mice were weighed daily; body condition scores and tumor volume were measured with calipers as 1/2×L×W×W and recorded and documented.

### 2.7 Safety profile analysis

To determine the safety of the c-MYC-mRNA drugs to the blood cells, serum electrolytes, kidney, liver, pancreatic, and gall bladder function were monitored. We collected blood and serum and sent them to the Memorial Sloan Kettering Cancer Core Pathology laboratory, which ran the full blood count, lipid profile, liver enzymes, kidney function, and electrolyte analyses. The reference is normal NSG mice. The analyst was blinded to the experimental details.

### 2.8 Pharmacokinetics of 3′UTRMYC1-18 *in vivo* in mice bearing tumors

The MYC-mRNA drug is complexed with the iron oxide nanocage. To understand the pharmacokinetics of the drug *in vivo* in the tumor-bearing mice, we administered 3′UTRMYC1-18 + IO nanocage (8.7 µg) intravenously; we collected the blood at 0 h, 3 h, 6 h, and 72 h and then obtained the serum. For each time point, we collected serum and then detected the iron oxide using an ELISA-based assay (Catalog #: HT20, Sigma Aldrich). We measured the absorbance intensity at various time points and presented the results. Our results demonstrate that 3′UTRMYC1-18 has a relatively stable half-life in the serum of greater than 24 h. The measurement of the iron oxide nanocage is a surrogate marker to easily determine the pharmacokinetics of 3′UTRMYC1-18 complexed with the iron oxide nanocage.

### 2.9 Biodistribution analysis

We previously described a Prussian blue stain approach that identified iron oxide nanocages in the tumors, livers, lungs, and brains of the tumor-bearing mice treated with the MYC-mRNA drug IO-nanocage complex in Awah et al. (2024).

### 2.10 Necropsy

Once the mice died, we took the fresh carcasses, dissected them, and collected the tumors, lungs, kidneys, livers, and brains. We rinsed the lungs in 1X PBS and took the images of the fresh organs. Subsequently, we collected fresh tissues of tumors and organs and froze them at −80°C. The tissues were then placed in 4% paraformaldehyde till they were sent to the pathology lab for paraffin embedding and hematoxylin and eosin (H&E) and immunohistochemistry (IHC) staining.

### 2.11 H&E staining of tumors and organs

The H&E images of the vector + nanocage (exp #1) and 3× IC50 groups were performed by the core pathology group of the Memorial Sloan Kettering Cancer Center. The H&E samples from the vector + nanocage (experiment #2), 6× IC50, and 9× IC50 groups were sectioned by QC and stained by JPD. The images of the stained tissues were obtained using an EVOS FL microscope at ×40 magnification, and quantification was done in ImageJ (RRID:SCR_003070) (https://imagej.net/ij/) and visualized in GraphPad Prism (v10) RRID:SCR_002798.

### 2.12 IHC staining of MYC and PD-L1 from tumors and organs

Briefly, to perform IHC staining against MYC and PD-L1 from the tumors and organs of the treated mice and the controls, we used the Abcam IHC protocol (https://www.abcam.com/en-us/technical-resources/protocols/ihc-with-samples-in-paraffin). We deparaffinized the tissue slides according to the protocol and performed enzymatic antigen retrieval using 1:1 trypsin concentrates and buffer. Washes were done with 1X TBST. Blocking was done with protein block (ab64212) for 1 h, after which the washes were done again. Next, we incubated the slides with primary antibodies against MYC (Anti-c-MYC 1:1000, cat no: 67,447-1-lg) or with PD-L1 (E1L3N^®^ XP^®^ Rabbit mAb #13684) overnight. Next day, the slides were washed with 1X TBST. The secondary antibody was added to the slide and incubated for 1 h. Subsequently, we washed the slides with 1x TBST and, to detect the signals, we used the DAB concentrate and enhanced the signal with enhancer. We counter-stained, mounting media was added, and sealed the slide with the cover slip . Images were obtained on an EVOS Fl at ×40. Target stains of the MYC and PD-L1 were quantified in ImageJ (https://imagej.net/ij/) RRID:SCR_003070.

### 2.13 Statistical analysis

The drug dose–response curve experiments to determine the c-MYC-mRNA downregulation by 3′UTRMYC1-18 were performed in multiple replicates and in a minimum number of N = 3. The *in vivo* dose-dependency experiment was performed in replicate with a minimum of four mice per group to validate therapeutic efficacy in the first experiment. In the second experiment, we used N = 3 mice per group, with an equal distribution of sexes (male and female and age and weight). A paired T-test was used to determine statistical significance between treated groups and controls. The Log Rank Mantel test was used to determine the statistical significance of survival differences between the *in vivo* treatment groups. The Kaplan–Meier survival curve was used to determine survival differences between the various treatment groups and controls. All data were plotted with GraphPad Prism (v.10) RRID:SCR_002798.

## 3 Results

### 3.1 Dose-dependent inhibition of lethal pancreatic cancer by 3′UTRMYC1-18

To validate the *in vitro* therapeutic efficacy of this novel c-MYC-mRNA drug (3′UTRMYC1-18), the metastatic drug-resistant pancreatic cancer cell lines MIA-PaCa-2, PSN1, and PANC1 were used. We performed a head-to-head, dose-dependent IC50 determination with 3′UTRMYC1-18 and the standard-of-care drugs, including a MYC-Max inhibitor (MYCi975). Figures 1A–C show that 3′UTRMYC1-18 achieved an IC50 of 1.98 µM and 1.2 µM, which is superior to these standard-of-care drugs and to MYCi975 in PSN1 and MIA-Paca2 but not in the PANC1 cells. The MYC-mRNA drug engaged the MYC-mRNA in a dose-dependent manner (Figure 1D), and we determined that the drug synergizes with epirubicin in a rational combination (Figure 1E).

To prove the safety of the engineered 3′UTRMYC1-18 to the normal healthy cells of the body, we performed a dose-dependent titration of the drug (3′UTRMYC1-18) in a head-to-head comparison with the standard-of-care drugs and MYCi975 in the normal cardiomyocytes, the AC16 cells, and the normal healthy epithelial cells, the RWPE1 (Supplementary Figures 1A, B). We found that 3′UTRMYC1-18 was not toxic to the normal cardiomyocytes and epithelial cells when compared to the standard-of-care drugs and MYCi975, which were toxic to the healthy cells (Supplementary Figures 1A, B). At the mRNA level, we show that 3′UTRMYC1-18 does not downregulate the MYC expression in healthy AC16 and RWPE1 cells compared to the controls (Supplementary Figures 1C, D).

**FIGURE 2:**
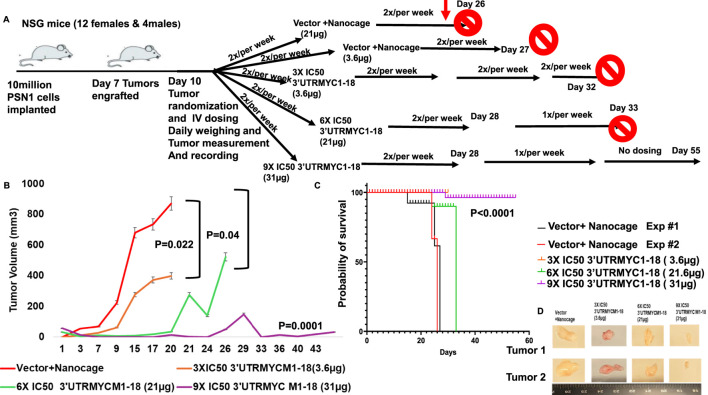
*In vivo* validation of 3′UTRMYC1-18 inhibition of lethal pancreatic cancer. **(A)** The schematic shows 10 million PSN1 cells implanted in the flanks of the NSG mice. After 7 days, they engrafted and, on day 10, they were randomized into vector + nanocage (21 µg), vector + nanocage (3.6 µg), 3× IC50 3′UTRMYC1-18+ nanocage (3.6 µg), 6× IC50 3′UTRMYC1-18 + nanocage, and 9× IC50 3′UTRMYC1-18 groups. Mice were dosed 2×/week till day 32 in the first experiment; blood was collected for safety and toxicity analysis on day 25. After day 28, mice were dosed 1×/week till day 33 in the 6× and 9× IC50 groups, and there was no further dosing in the 9× IC50 group until the end on day 55. **(B)** The chart shows the daily tumor volume measurement of the vector + nanocage (#1-red), vector + nanocage (#2-black), 3× IC50 3′UTRMYC1-18 (orange), 6× IC50 3′UTRMYC1-18 (green), and 9× IC50 3′UTRMYC1-18 (purple) groups. **(C)** The Kaplan–Meier chart shows the survival of groups of tumor-bearing mice treated with the vector + nanocage and the dose-dependent 3′UTRMYC1-18 treatments. ***P < 0.0001. **(D)** Images of two tumors from the vector + nanocage, the 3× IC50, 6× IC50, and 9× IC50 3′UTRMYC1-18 groups. ****p < 0.00001, ***p = 0.0001, *p = 0.02, and *p = 0.044, Two-tailed T-test.

To prove the *in vivo* efficacy of the drug, we performed two independent experiments (Figure 2A), both titrating the drug in a dose-dependent manner. The tumor-bearing mice treated with the c-MYC drugs (3.6 µg) show a significant reduction in the tumor volume (*p = 0.019) compared to the controls (Figures 2B,C), with a tumor volume reduction of approximately half (Figure 2B). They also survived better than the vector + nanocage-treated group (*p = 0.0117) (Figure 2C).

To prove that the c-MYC-mRNA drug inhibited c-MYC *in vivo* in a titratable dose-dependent manner in lethal pancreatic cancer, we titrated the dose of the MYC-mRNA drug from the first *in vivo* experiment of 3× IC50 (3.6 µg) to 6× IC50 (21.6 µg) and 9× IC50 (32 µg). The control was the vector + nanocage-treated group (Figure 2B). The 9× IC50 group achieved complete *in vivo* inhibition of pancreatic cancer (Figures 2B,D), with an exceptionally significant survival outcome (Figure 2C), ****P < 0.0001.

### 3.2 3′UTRMYC1-18 achieved on-target inhibition of the pancreatic tumor by downregulating c-MYC and PD-L1

**FIGURE 3:**
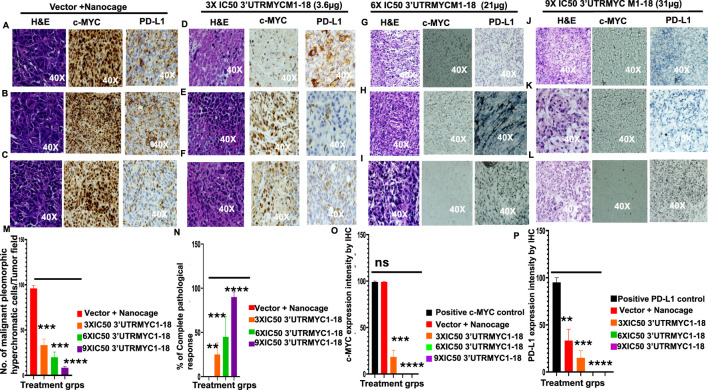
MYC mRNA therapeutically downregulates malignant cancer tissue, c-MYC, and PD-L1 expression to inhibit pancreatic cancer. **(A)** Images show the H&E, c-MYC, and PD-L1 IHC stains of tumor 1 from the vector + nanocage group. (N = 3 vector + nanocage). **(B)** Images show the H&E, c-MYC, and PD-L1 IHC stains of tumor 2 from the vector + nanocage group. (N = 3 vector + nanocage). **(C)** Images show the H&E, c-MYC, and PD-L1 IHC stains of tumor 3 from the vector + nanocage group. (N = 3 vector + nanocage). **(D)** Images show the H&E, c-MYC, and PD-L1 IHC stains of tumor 1 from the 3× IC50 3′UTRMYC1-18 group. (N = 3). **(E)** Images show the H&E, c-MYC, and PD-L1 IHC stains of tumor 2 from the 3× IC50 3′UTRMYC1-18 group. (N = 3). **(F)** Images show the H&E, c-MYC, and PD-L1 IHC stains of tumor 3 from the 3× IC50 3′UTRMYC1-18 group. (N = 3). **(G)** Images show the H&E, c-MYC, and PD-L1 IHC stains of tumor 1 from the 6× IC50 3′UTRMYC1-18 group. (N = 3). **(H)** Images show the H&E, c-MYC, and PD-L1 IHC stains of tumor 2 from the 6× IC50 3′UTRMYC1-18 group. (N = 3). **(I)** Images show the H&E, c-MYC, and PD-L1 IHC stains of tumor 3 from the 6× IC50 3′UTRMYC1-18 group. (N = 3). **(J)** Images show the H&E, c-MYC, and PD-L1 IHC stains of tumor 1 from the 9× IC50 3′UTRMYC1-18. (N = 3) group. **(K)** Images show the H&E, c-MYC, and PD-L1 IHC stains of tumor 2 from the 9× IC50 3′UTRMYC1-18 group. (N = 3). **(L)** Images show the H&E, c-MYC, and PD-L1 IHC stains of tumor 3 from the 9× IC50 3′UTRMYC1-18 group. (N = 3). **(M)** Bar charts show the quantification of the number of malignant pleomorphic hyperchromatic cells in the tumors from the vector + nanocage, 3× IC50, 6× IC50, and 9× IC50 3′UTRMYC1-18 groups. (N = 3 vector + nanocage, N = 3 3× IC50 3′UTRMYC1-18, N = 3 6× IC50 3′UTRMYC1-18, N = 3 9× IC50 3′UTRMYC1-18). **(N)** Bar charts show the percentage of complete pathological responses in the tumors from the vector + nanocage, 3× IC50, 6× IC50, and 9× IC50 3′UTRMYC1-18 groups. (N = 3 vector + nanocage, N = 3 3× IC50 3′UTRMYC1-18, N = 3 6× IC50 3′UTRMYC1-18, N = 3 9× IC50 3′UTRMYC1-18). **(O)** The bar chart shows the c-MYC expression by IHC in the positive control and in the tumors from the vector + nanocage, 3× IC50, 6× IC50, and 9× IC50 3′UTRMYC1-18 groups. (N = 3 Vector + Nanocage, N = 3 3× IC50 3′UTRMYC1-18, N = 3 6× IC50 3′UTRMYC1-18, N = 3 9× IC50 3′UTRMYC1-18). **(P)** The bar chart shows the PD-L1 expression by IHC in the positive control and the tumors from the vector + nanocage, 3× IC50, 6× IC50, and 9× IC50 3′UTRMYC1-18 groups. (N = 3 vector + nanocage, N = 3 3× IC50 3′UTRMYC1-18, N = 3 6× IC50 3′UTRMYC1-18, N = 3 9× IC50 3′UTRMYC1-18). ****p < 0.00001, ***p = 0.0002, **p = 0.0012, p = ns (non-significant), Two-tailed T-test.

To confirm that the MYC-mRNA drug achieved on-target inhibition of c-MYC in the primary tumor to achieve therapeutic efficacy, we performed H&E staining of the tumors (3) from the vector + nanocage, the 3× IC50, 6× IC50, and 9× IC50-treated tumor-bearing mice. We found that the vector + nanocage-treated tumors were hyperchromatic, malignant pleomorphic cells (Figures 3A–C). The 3× IC50 dose achieved complete pathological response in 1/3 and partial response in 2/3 of the tumors (Figures 3D–F). The 6× IC50 dose achieved complete pathological response in 1/3, partial response in 1/3, and no response in 1/3 of the tumors (Figures 3G–I). The 9× IC50 dose achieved 100% complete pathological response (3/3) (Figures 3J–L), quantified in Figures 3M,N. Next, we stained for c-MYC expression by IHC in three tumors from each group. We found a very high level of c-MYC in the vector + nanocage-treated group (Figures 3A–C), a moderate level of c-MYC expression in the 3× IC50-treated tumor group (Figures 3D–F), and complete inhibition of the c-MYC expression in the 6× IC50 and 9× IC50 3′UTRMYC1-18-treated groups (Figures 3G–J,L), quantified in Figure 3O. We found similar dose-dependent downregulation for PD-L1 expression in the same tumors with dose-dependent downregulation of the c-MYC expression (Figures 3A–L), quantified in Figure 3P.

Taken together, these data demonstrate that the c-MYC-mRNA drug achieved dose-dependent, on-target inhibition of the lethal pancreatic tumors via dose-dependent downregulation of the c-MYC and PD-L1 expression.

### 3.3 Downregulation of c-MYC inhibited liver, lung, and pancreatic cancer metastases

**FIGURE 4:**
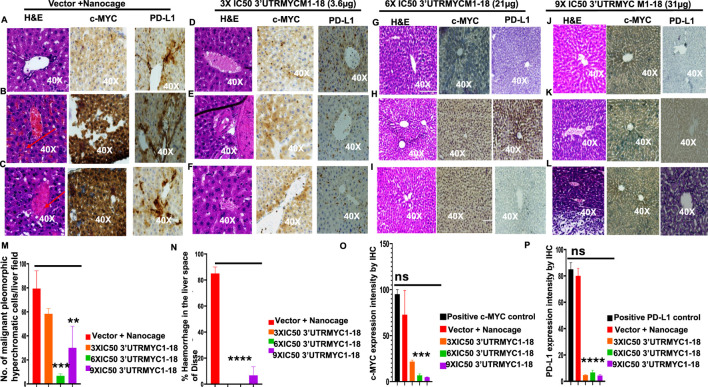
3′UTRMYC1-18 inhibits liver metastasis in pancreatic cancer by downregulating c-MYC and PD-L1 expression. **(A)** Images show the H&E, c-MYC, and PD-L1 IHC stains of liver 1 from the vector + nanocage group. (N = 3 vector + nanocage). **(B)** Images show the H&E, c-MYC, and PD-L1 IHC stains of liver 2 from the vector + nanocage group. (N = 3 vector + nanocage). Hemorrhagic lesions are marked with a red arrow. **(C)** Images show the H&E, c-MYC, and PD-L1 IHC stains of liver 3 from the vector + nanocage group. (N = 3 Vector + Nanocage). Hemorrhagic lesions are marked with a red arrow. **(D)** Images show the H&E, c-MYC, and PD-L1 IHC stains of liver 1 from the 3× IC50 3′UTRMYC1-18 group. (N = 3). **(E)** Images show the H&E, c-MYC, and PD-L1 IHC stains of liver 2 from the 3× IC50 3′UTRMYC1-18 group. (N = 3). Hemorrhagic lesions are marked with a red arrow. **(F)** Images show the H&E, c-MYC, and PD-L1 IHC stains of liver 3 from the 3× IC50 3′UTRMYC1-18 group. (N = 3). **(G)** Images show the H&E, c-MYC, and PD-L1 IHC stains of liver 1 from the 6× IC50 3′UTRMYC1-18. (N = 3) group. **(H)** Images show the H&E, c-MYC, and PD-L1 IHC stains of liver 2 from the 6× IC50 3′UTRMYC1-18 group. (N = 3). **(I)** Images show the H&E, c-MYC, and PD-L1 IHC stains of liver 3 from the 6× IC50 3′UTRMYC1-18 group. (N = 3). **(J)** Images show the H&E, c-MYC, and PD-L1 IHC stains of liver 1 from the 9× IC50 3′UTRMYC1-18 group. (N = 3). **(K)** Images show the H&E, c-MYC, and PD-L1 IHC stains of liver 2 from the 9× IC50 3′UTRMYC1-18 group. (N = 3). **(L)** Images show the H&E, c-MYC, and PD-L1 IHC stains of liver 3 from the 9× IC50 3′UTRMYC1-18 group. (N = 3). **(M)** Bar charts show the quantification of the number of malignant pleomorphic hyperchromatic cells in the livers from the vector + nanocage, 3× IC50, 6× IC50, and 9× IC50 3′UTRMYC1-18 groups. (N = 3 vector + nanocage, N = 3 3× IC50 3′UTRMYC1-18, N = 3 6× IC50 3′UTRMYC1-18, N = 3 9× IC50 3′UTRMYC1-18). **(N)** Bar charts show the percentage of hemorrhagic lesions in the livers from the vector + nanocage, 3× IC50, 6× IC50, and 9× IC50 3′UTRMYC1-18 groups. (N = 3 vector + nanocage, N = 3 3× IC50 3′UTRMYC1-18, N = 3 6× IC50 3′UTRMYC1-18, N = 3 9× IC50 3′UTRMYC1-18). **(O)** The bar chart shows the c-MYC expression by IHC in the positive control and the livers from the vector + nanocage, 3× IC50, 6× IC50, and 9× IC50 3′UTRMYC1-18 groups. (N = 3 vector + nanocage, N = 3 3× IC50 3′UTRMYC1-18, N = 3 6× IC50 3′UTRMYC1-18, N = 3 9× IC50 3′UTRMYC1-18). **(P)** The bar chart shows the PD-L1 expression by IHC in the positive control and in the livers from the vector + nanocage, 3× IC50, 6× IC50, and 9× IC50 3′UTRMYC1-18 groups. (N = 3 vector + nanocage, N = 3 3× IC50 3′UTRMYC1-18, N = 3 6× IC50 3′UTRMYC1-18, N = 3 9× IC50 3′UTRMYC1-18). ****p < 0.000025, ***p = 0.00018, **p = 0.004, p = ns (non-significant), Two-tailed T-test.

The liver is the most common site of pancreatic cancer metastasis. We investigated whether inhibition of the tumor and downregulation of the c-MYC and PD-L1 expression led to the inhibition of metastasis to the lungs, liver, and pancreas. We performed H&E staining of the three livers from the vector + nanocage, 3× IC50, 6× IC50, and 9× IC50 groups of the 3′UTRMYC1-18-treated tumor-bearing mice (Figure 4). We found hyperchromatic pleomorphic cells (3/3), perforated (3/3), and eosinophilic hemorrhagic lesions (2/3) in the livers of the vector + nanocage-treated tumor-bearing mice (Figures 4A–C). The 3× IC50 3′UTRMYC-18 dose shows a lesser level of hyperchromatic pleomorphic cells (3/3), few perforations (3/3), and no eosinophilic hemorrhagic lesions (0/3) in the livers of the treated tumor-bearing mice (Figures 4D–F). The 6× IC50 3′UTRMYC1-18 dose achieved more than 75% inhibition of the hyperchromatic pleomorphic cells (3/3), no perforations (0/3), and no eosinophilic hemorrhagic lesions (0/3) in the livers of the tumor-bearing mice treated (Figures 4G–I). The 9× IC50 3′UTRMYC1-18 dose achieved (1/3) inhibition of the hyperchromatic pleomorphic cells, (1/3) perforation, and hemorrhagic lesion in the liver of the tumor-bearing mice (Figures 4J–L). The quantification of the hyperchromatic pleomorphic cells, the liver architecture preservation, and hemorrhagic lesions are shown in Figures 4M,N. We stained for the c-MYC and PD-L1 expression in the control and different treatment groups. We found markedly elevated levels of c-MYC (2/3) and PD-L1 (3/3) in the livers treated with the vector + nanocage (Figures 4A–C) and moderate expression levels of c-MYC and PD-L1 in the 3× IC50 dose-treated group (Figures 4D–F). In the 6× IC50 and 9× IC50 groups, there is complete downregulation of the c-MYC in the liver (Figures 4G–L). We found only (1/3) liver in the 6× IC50 and 9× IC50 with a markedly elevated PD-L1 expression (Figures 4G–L), quantified in Figures 4O,P. Taken together, we show the dose-dependent inhibition of liver metastasis through the downregulation of the c-MYC and PD-L1.

**FIGURE 5:**
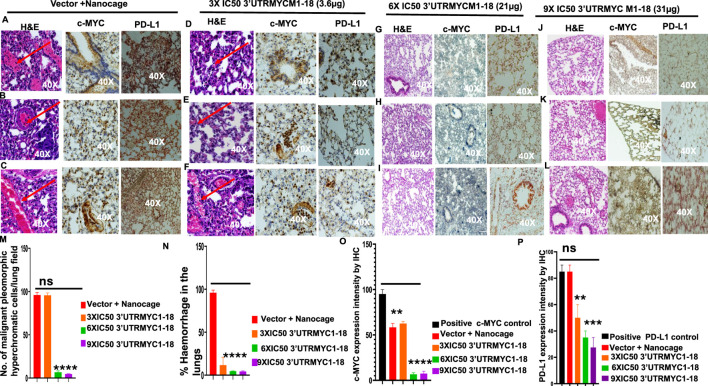
The inhibition of lung metastasis in pancreatic cancer through c-MYC and PD-L1 expression downregulation by the c-MYC-mRNA drug. **(A)** Images show the H&E, c-MYC, and PD-L1 IHC stains of lung 1 from the vector + nanocage group. (N = 3 Vector + Nanocage). Hemorrhagic lesions are marked with a red arrow. **(B)** Images show the H&E, c-MYC, and PD-L1 IHC stains of lung 2 from the vector + nanocage group. (N = 3 Vector + Nanocage). A hemorrhagic lesion is marked with a red arrow. **(C)** Images show the H&E, c-MYC, and PD-L1 IHC stains of lung 3 from the vector + nanocage group. (N = 3 Vector + Nanocage). A hemorrhagic lesion is marked with a red arrow. **(D)** Images show the H&E, c-MYC, and PD-L1 IHC stains of lung 1 from the 3× IC50 3′UTRMYC1-18 group. (N = 3). A hemorrhagic lesion is marked with a red arrow. **(E)** Images show the H&E, c-MYC, and PD-L1 IHC stains of lung 2 from the 3× IC50 3′UTRMYC1-18 group. (N = 3). A hemorrhagic lesion is marked with a red arrow. **(F)** Images show the H&E, c-MYC, and PD-L1 IHC stains of lung 3 from the 3× IC50 3′UTRMYC1-18 group. (N = 3). A hemorrhagic lesion is marked with a red arrow. **(G)** Images show the H&E, c-MYC, and PD-L1 IHC stains of lung 1 from the 6× IC50 3′UTRMYC1-18 group. (N = 3). **(H)** Images show the H&E, c-MYC, and PD-L1 IHC stains of lung 2 from the 6× IC50 3′UTRMYC1-18 group. (N = 3). **(I)** Images show the H&E, c-MYC, and PD-L1 IHC stains of lung 3 from the 6× IC50 3′UTRMYC1-18 group. (N = 3). **(J)** Images show the H&E, c-MYC, and PD-L1 IHC stains of lung 1 from the 9× IC50 3′UTRMYC1-18 group. (N = 3). **(K)** Images show the H&E, c-MYC, and PD-L1 IHC stains of lung 2 from the 9× IC50 3′UTRMYC1-18 group. (N = 3). **(L)** Images show the H&E, c-MYC, and PD-L1 IHC stains of lung 3 from the 9× IC50 3′UTRMYC1-18 group. (N = 3). **(M)** Bar charts show the quantification of the number of malignant pleomorphic hyperchromatic cells in the lungs from the vector + nanocage, 3× IC50, 6× IC50, and 9× IC50 3′UTRMYC1-18 groups. (N = 3 vector + nanocage, N = 3 3× IC50 3′UTRMYC1-18, N = 3 6× IC50 3′UTRMYC1-18, N = 3 9× IC50 3′UTRMYC1-18). **(N)** Bar charts show the percentage of hemorrhagic lesions in the lungs from the vector + nanocage, 3× IC50, 6× IC50, and 9× IC50 3′UTRMYC1-18 groups. (N = 3 vector + nanocage, N = 3 3× IC50 3′UTRMYC1-18, N = 3 6× IC50 3′UTRMYC1-18, N = 3 9× IC50 3′UTRMYC1-18). **(O)** The bar chart shows the c-MYC expression by IHC in the positive control and in the lungs from the vector + nanocage, 3× IC50, 6× IC50, and 9× IC50 3′UTRMYC1-18 groups. (N = 3 vector + nanocage, N = 3 3× IC50 3′UTRMYC1-18, N = 3 6× IC50 3′UTRMYC1-18, N = 3 9× IC50 3′UTRMYC1-18). **(P)** The bar chart shows the PD-L1 expression by IHC in the positive control and in the lungs from the vector + nanocage, 3× IC50, 6× IC50, and 9× IC50 3′UTRMYC1-18 groups. (N = 3 vector + nanocage, N = 3 3× IC50 3′UTRMYC1-18, N = 3 6× IC50 3′UTRMYC1-18, N = 3 9× IC50 3′UTRMYC1-18). P = ns (non-significant), ***p = 0.000105, **p = 0.002, Two-tailed T-test.

Next, we investigated the lungs to understand whether 3′UTRMYC1-18 inhibited pancreatic cancer metastasis to the lungs. We performed H&E staining of the lungs of the tumor-bearing mice in the vector + nanocage, 3× IC50, 6× IC50, and 9× IC50 3′UTRMYC1-18 groups. We found hyperchromatic, pleomorphic cells (3/3), and eosinophilic hemorrhagic lesions (3/3) in the lungs treated with the vector + nanocage (Figures 5A–C). In the 3× IC50 3′UTRMYC1-18-treated mice, we found malignant hyperchromatic pleomorphic cells (3/3) and (1/3) eosinophilic hemorrhagic lesions in the lungs (Figures 5D–F). In the 6× IC50 and 9× IC50 3′UTRMYC1-18-treated lungs, we found complete inhibition (3/3) of the malignant pleomorphic cells and no eosinophilic hemorrhagic cells in the lungs (Figures 5G–L). We quantified these data in Figures 5M,N.

We extended our analysis into the c-MYC and PD-L1 expression downregulation by comparing the expression of the c-MYC and PD-L1 in the lungs of the vector + nanocage-treated tumor-bearing mice and the dose-dependent treatment groups (Figures 5A–C). We found a clear dose-dependent downregulation of the c-MYC and PD-L1 in the lungs treated with 3×, 6×, and 9× IC50 3′UTRMYC1-18 (Figures 5D–K). The data are quantified in Figures 5O,P. Taken together, we demonstrate that the drug achieved distant organ lung metastasis inhibition by downregulating the c-MYC and PD-L1 in a dose-dependent manner.

In the brain cells, we found the inhibition of brain metastasis in the pancreatic cancer tumor-bearing mice in the 6× and the 9× IC50 3′UTRMYC1-18 groups (Supplementary Figures 2C, D) compared to the controls, the vector + nanocage, and the 3× IC50 3′UTRMYC1-18 groups (Supplementary Figures 2A, B). The inhibition of brain metastasis is dose dependent in the order 9× IC50 = 6× IC50 > 3× IC50 3′UTRMYC1-18 > vector + nanocage-treated groups (Supplementary Figures 2A-D), quantified in Supplementary Figure S2E.

### 3.4 *In vivo* safety and toxicity profiles and pharmacokinetics analysis show that 3′UTRMYC1-18 is safe for blood cells

**FIGURE 6:**
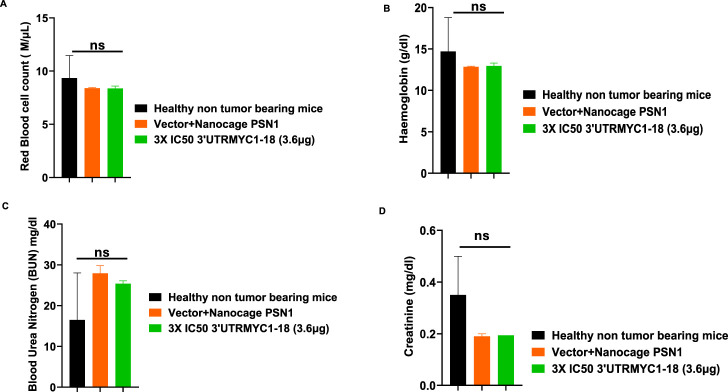
The safety profile of 3′UTRMYC1-18 on the red blood cells and kidney function in pancreatic cancer tumor-bearing mice and controls. **(A)** The bar chart shows the red blood cell count in the healthy non-tumor-bearing mice and the tumor-bearing mice from the vector + nanocage (N = 2) and 3× IC50 3′UTRMYC1-18 (N = 2) groups. **(B)** The bar chart shows the hemoglobin levels in the healthy non-tumor-bearing mice and the tumor-bearing mice from the vector + nanocage (N = 2) and the 3× IC50 3′UTRMYC1-18 (N = 2) groups. **(C)** The bar chart shows the blood urea nitrogen levels in the healthy non-tumor-bearing mice and the tumor-bearing mice from the vector + nanocage (N = 2) and the 3× IC50 3′UTRMYC1-18 (N = 2) groups. **(D)** The bar chart shows the creatinine levels in the healthy non-tumor-bearing mice and the tumor-bearing mice from the vector + nanocage (N = 2) and the 3× IC50 3′UTRMYC1-18 (N = 2) groups. p = ns (non-significant), Two-tailed T-test.

**FIGURE 7:**
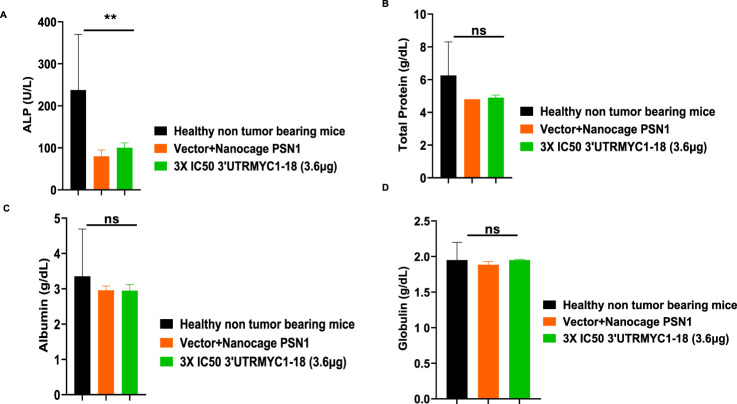
The safety profile of 3′UTRMYC1-18 on the liver enzyme ALP, total protein, albumin, and albumin in pancreatic cancer tumor-bearing mice and controls. **(A)** The bar chart shows the ALP (alkaline phosphatase) levels in the healthy non-tumor-bearing mice, in the tumor-bearing mice from the vector + nanocage (N = 2), and in the 3× IC50 3′UTRMYC1-18 (N = 2) groups. **(B)** The bar chart shows the total protein levels in the healthy non-tumor-bearing mice and the tumor-bearing mice from the vector + nanocage (N = 2) and the 3× IC50 3′UTRMYC1-18 groups (N = 2). **(C)** The bar chart shows the albumin levels in the healthy non-tumor-bearing mice and the tumor-bearing mice from the vector + nanocage (N = 2) and the 3× IC50 3′UTRMYC1-18 groups (N = 2). **(D)** The bar chart shows the globulin levels in the healthy non-tumor-bearing mice and the tumor-bearing mice from the vector + nanocage (N = 2) and the 3× IC50 3′UTRMYC1-18 groups (N = 2). **p = 0.023, p = ns (non-significant), Two-tailed T-test.

**FIGURE 8:**
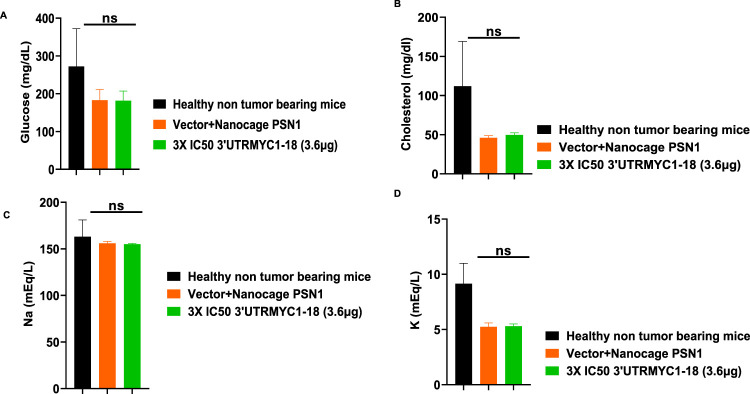
The safety profile of 3′UTRMYC1-18 on the pancreatic function, glucose, cholesterol, and electrolytes in pancreatic cancer tumor-bearing mice and controls. **(A)** The bar chart shows the glucose levels in the healthy non-tumor-bearing mice and the tumor-bearing mice from the vector + nanocage (N = 2) and the 3× IC50 3′UTRMYC1-18 groups (N = 2). **(B)** The bar chart shows the cholesterol levels in the healthy non-tumor-bearing mice and the tumor-bearing mice from the vector + nanocage (N = 2) and the 3× IC50 3′UTRMYC1-18 groups (N = 2). **(C)** The bar chart shows the sodium levels in the healthy non-tumor-bearing mice and the tumor-bearing mice from the vector + nanocage (N = 2) and the 3× IC50 3′UTRMYC1-18 groups (N = 2). **(D)** The bar chart shows the potassium levels in the healthy non-tumor-bearing mice and the tumor-bearing mice from the vector + nanocage (N = 2) and the 3× IC50 3′UTRMYC1-18 groups (N = 2). p = ns (non-significant), Two-tailed T-test.

To prove the MYC-mRNA drug safety in the tumor-bearing mice compared to healthy controls, we collected blood on day 25 (Figure 1A) to assay the blood cells, electrolytes, liver enzymes, kidney function, and pancreatic and biliary function analysis from the 3× IC50 group (Supplementary Table S1, Supplementary Table S2). We found no change in red blood cells (Figure 6A), no change in hemoglobulin (Figure 6B) and other blood indices (see Supplementary Table S1), no change in the levels of the blood urea nitrogen levels (Figure 6C), and no change in the weight of the mice that received the dose-dependent mRNA drugs (Supplementary Figure S3A). We found a normal range of creatinine levels (Figure 6D). We examined the liver ALP levels (Figure 7A) and other liver enzyme levels (Supplementary Table S2). We found that all levels were reduced except for the AST levels, which were elevated in one of the tumor-bearing mice treated with 3× IC50 3′UTRMYC1-18 (Supplementary Table S2). The total protein, albumin, and globulin levels were within the same range (Figures 7B–D). The glucose levels, the cholesterol levels, and the electrolytes (Na and K) levels are all within the normal ranges (Figures 8A–D) and Supplementary Table S2. Next, we performed pharmacokinetics analysis of the MYC-mRNA drug in the serum of the tumor-bearing mice treated with the drug. We found that the drug has a stable half-life in the serum of greater than 24 h (Supplementary Figure S4A).

These data demonstrate that the drug is safe, stable, and well tolerated with no blood dyscrasia, no electrolyte imbalance, no kidney failure, no liver failure, and no pancreatic enzyme function abnormalities.

## 4 Discussion

We demonstrated that lethal pancreatic cancer driven by c-MYC can be inhibited with the c-MYC-mRNA drug, 3′UTRMYC1-18, in a dose-dependent, titratable manner to achieve a remarkably significant survival outcome. The drug was safe with a relatively stable long half-life and well tolerated with no evidence of blood dyscrasia, electrolyte imbalance, kidney, liver, and pancreatic enzyme abnormalities for the 3× IC50 dose tested in the safety and toxicology assays.

The drug achieved a dose-dependent significant survival outcome. The 3× IC50 and 6× IC50 groups achieved a 6-day survival difference compared to the control. When we increased the dose to 9× IC50, we achieved a 28-day survival difference when compared to the control. These data suggest that the lethal pancreatic cancer can indeed be inhibited *in vivo* with very significant survival outcomes and a safe profile by 3′UTRMYC1-18, thus serving a strong rationale for acceleration of the novel c-MYC-mRNA drug toward a phase 1 trial in humans with diverse c-MYC-driven cancers, including lethal pancreatic cancers of all stages.

We have demonstrated the generalizability of the MYC-mRNA drug’s therapeutic efficacy across different pancreatic cancer models of PSN1, MIA-Paca-2, and PANC1 and validated its inhibition of the high c-MYC-expressing PSN1 *in vivo* with significant survival outcome. However, the model used is an NSG metastatic xenograft model. This work will be extended in a genetically modified mouse model (GEMM) of pancreatic cancer. This will give insight into how the immune cells and inhibition of the checkpoint proteins support the therapeutic efficacy of the ribosome.

## Data Availability

The original contributions presented in the study are included in the article/Supplementary Material, further inquiries can be directed to the corresponding author.

## Supplementary material

SUPPLEMENTARY FIGURE S1 Safety of 3′UTRMYC1-18 in the normal cardiomyocytes and epithelial cells. **(A)** Dose–response curve of 3′UTRMYC1-18 and standard-of-care drugs and MYCi975 in AC16. **(B)** Dose–response curve of 3′UTRMYC1-18 and standard-of-care drugs and MYCi975 in AC16. **(C)** The bar chart shows MYC expression in AC16 cells treated in a dose-dependent manner with 3′UTRMYC1-18. **(D)** Bar chart shows MYC expression in RWPE1 cells treated in a dose-dependent manner with 3′UTRMYC1-18.

SUPPLEMENTARY FIGURE S2 H&E images of the brain of the pancreatic cancer-treated mice and controls. **(A)** H&E images of brains 1–3 from the vector + nanocage-treated group. The brain metastasis lesions are marked with a red arrow. **(B)** H&E images of brains 1–3 from the 3× IC50 3′UTRMYC1-18 group. The brain metastasis lesions are marked with a red arrow. **(C)** H&E images of brains 1–3 from the 6× IC50 3′UTRMYC1-18 group. **(D)** H&E images of brains 1–3 from the 9× IC50 3′UTRMYC1-18-treated group. **(E)** The bar chart shows the quantification of the brain metastases in brains of tumor-bearing mice from the vector + nanocage, 3× IC50, 6× IC50, and 9× IC50 3′UTRMYC1-18 groups. P = ns (non-significant), ****p < 0.0003, Two-tailed T-test.

SUPPLEMENTARY FIGURE S3 Daily weight recording of the pancreatic cancer-treated mice and controls. **(A)** Chart shows the daily weight recording of the tumor-bearing mice from the vector + nanocage, 3× IC50, 6× IC50, and 9× IC50 3′UTRMYC1-18 groups.

SUPPLEMENTARY FIGURE S4 The pharmacokinetics of 3′UTRMYC1-18 in tumor-bearing mice. **(A)** The chart shows the time-dependent absorbance of the 3′UTRMYC-18+IO nanocage in the serum of the tumor-bearing mice.

SUPPLEMENTARY TABLE S1 Complete blood profile analysis, red blood cell and platelet indices, and automated differentials.

SUPPLEMENTARY TABLE S2 Electrolyte and liver, kidney, pancreatic, and gall bladder function analysis.
