# Supplementary figures and images for: c-MYC mRNA destabilization inhibited lethal pancreatic cancer in vivo with significant survival outcomes
Source: Front Pharmacol. 2025 Aug 29;16:1630476. doi: 10.3389/fphar.2025.1630476 (PMC12426058; doi:10.3389/fphar.2025.1630476)

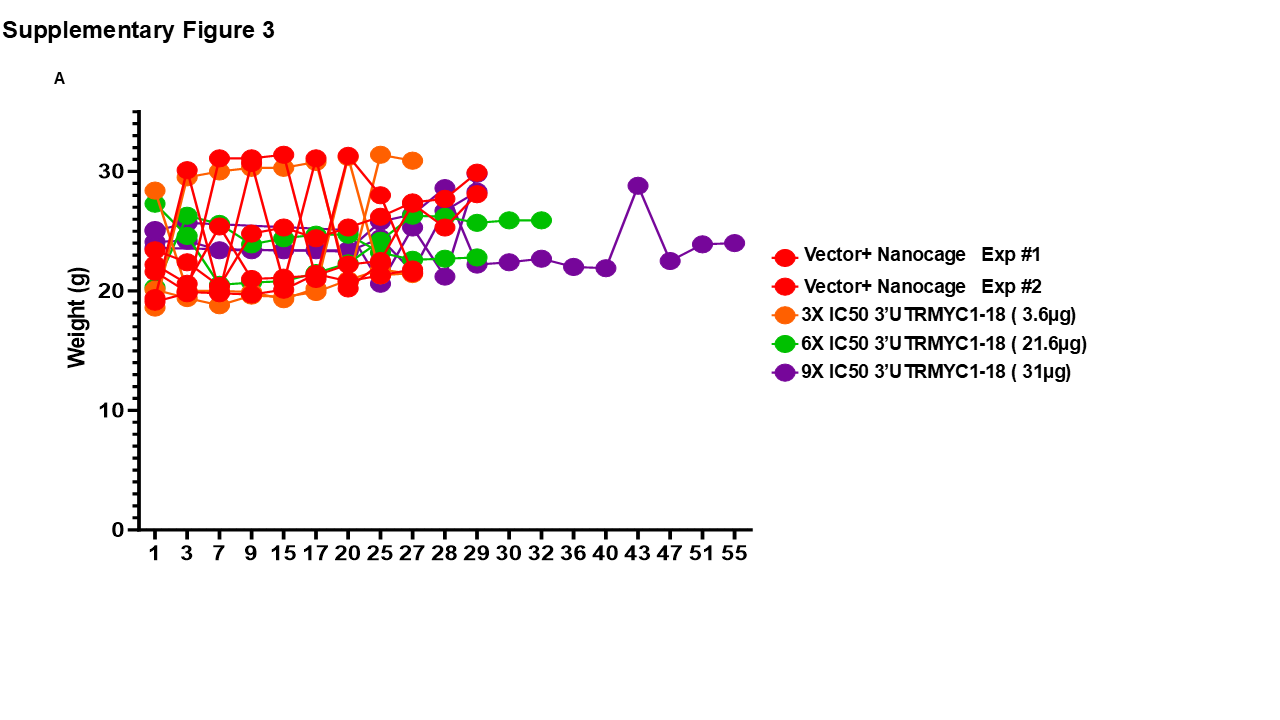

Supplement: Supplementary file 2 [file Image3.tif]

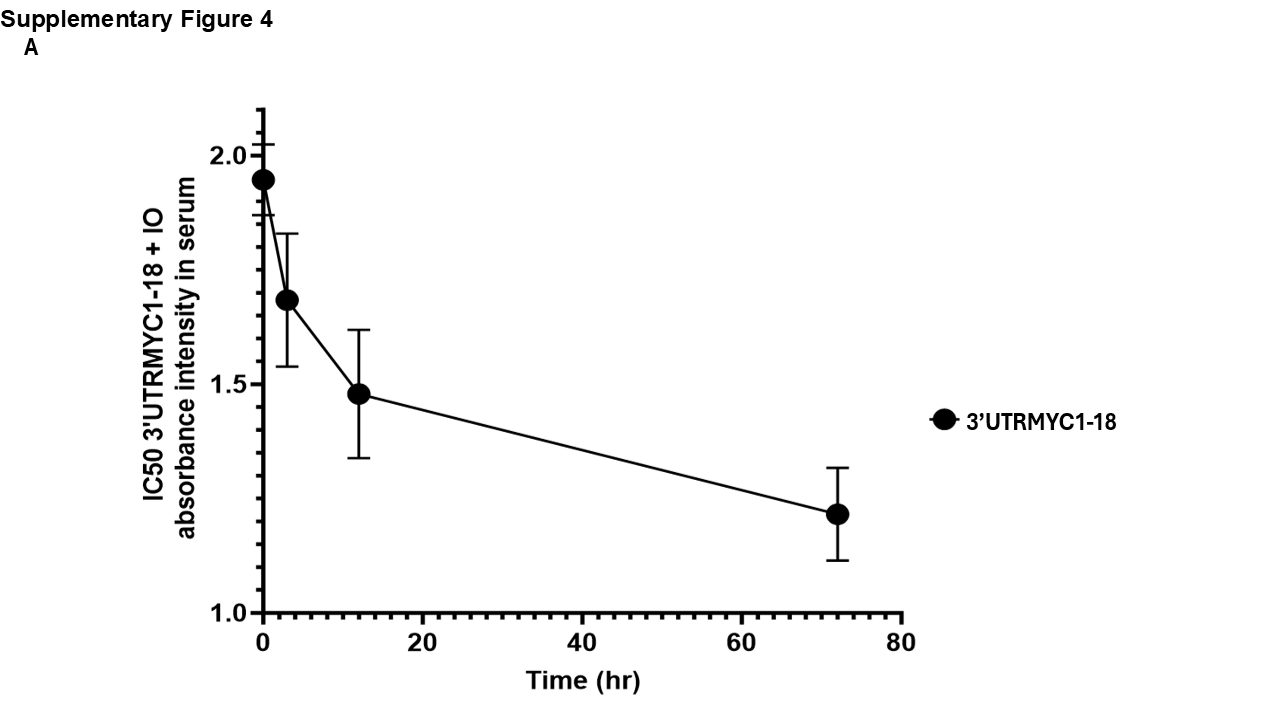

Supplement: Supplementary file 3 [file Image4.tif]

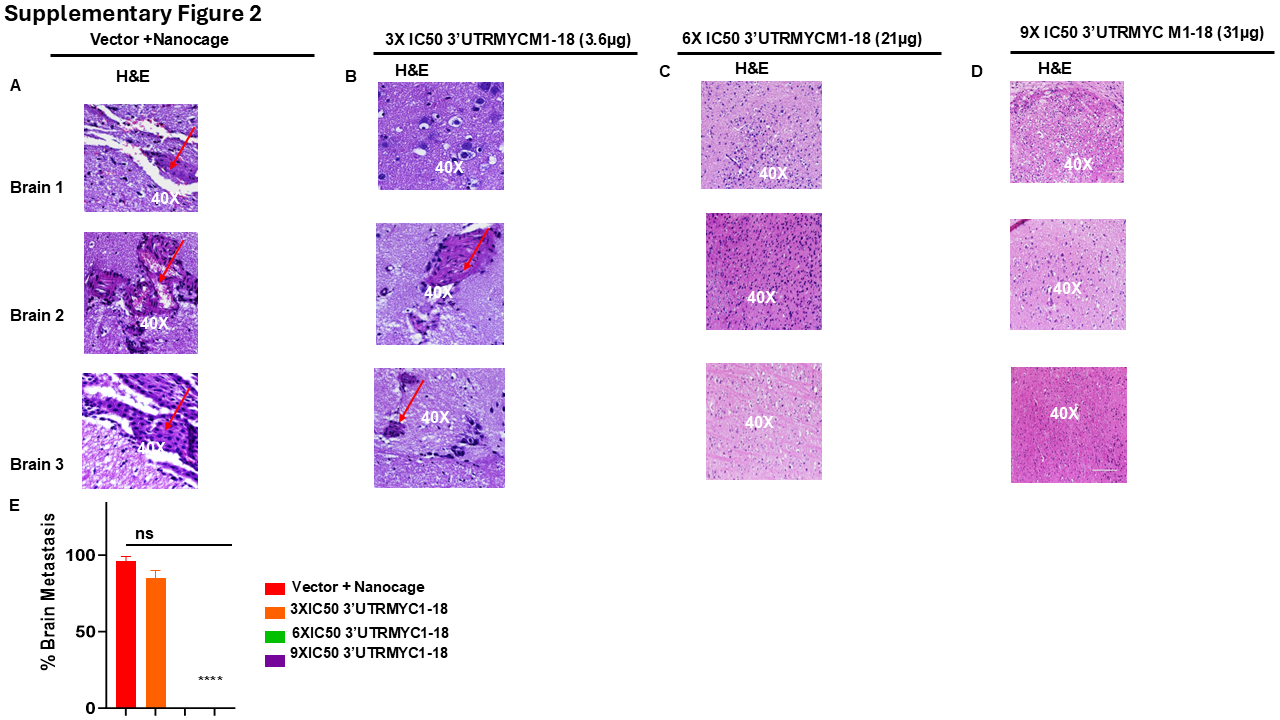

Supplement: Supplementary file 4 [file Image2.tif]

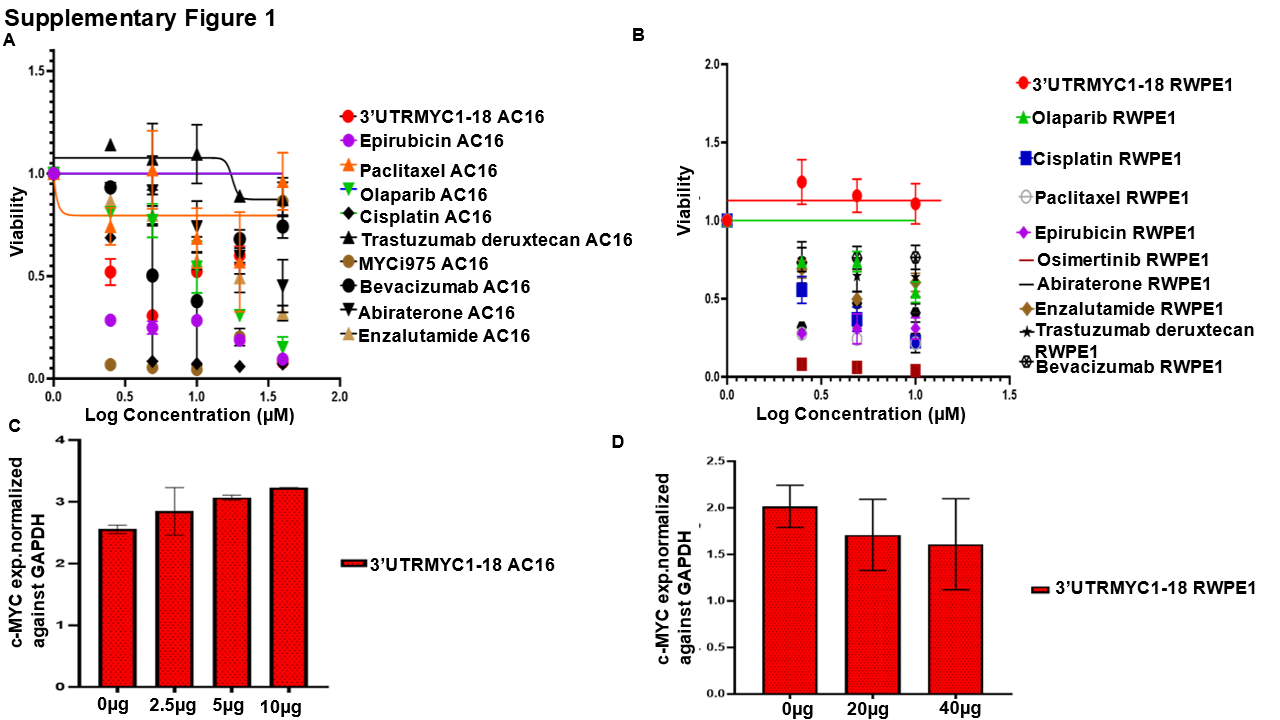

Supplement: Supplementary file 5 [file Image1.tif]
